# Chronic Cervical Catarrh of the Uterus

**Published:** 1900-10-27

**Authors:** George A. Hawkins-Ambler

**Affiliations:** Surgeon to the Samaritan Hospital for Women, Assistant-Surgeon, Stanley Hospital, Liverpool


### Hospital Clinics and Medical Progress.

### CHRONIC CERVICAL CATARRH OF THE UTERUS.

By George A. Hawkins-Ambler, F.R.C.S.E., Surgeon to the Samaritan Hospital for Women, Assistant-Surgeon, Stanley Hospital, Liverpool.

Endocervicitis, while one of the commonest gynæcological lesions the practitioner will meet with, is more accessible to him than most other diseases of the genital tract, and is usually curable by judicious treatment if adopted early. Nevertheless, we too often see cervical catarrh that has become aggravated by neglect, or by treatment adopted with more enthusiasm than with judgment tempered by a due appreciation of the underlying pathological conditions and the many factors that may combine to produce or complicate it. The constitutional origin of the disease, e.g., is frequently overlooked, and this accounts for many disappointments in practice. It results in a great deal of aggressive local tinkering, which is explained by our modern tendency to fix the attention on obvious local conditions, to the neglect of general considerations of at least equal importance—a tendency not by any means confined to gynæcologists. We have all seen how often the affection accompanies constitutional derangements and disappears as they are remedied; and we have seen, too, how frequently attention has been limited to general symptoms, while the local trouble has been allowed to go on unchecked. Improvement in general health has waited on the cure of the cervical catarrh, and thenceforward keeps pace with it. I am quite satisfied that, whatever value we attach to local treatment, we cannot afford to neglect the constitutional condition of our patients. Thus we see too much cervical catarrh in scrofulous and anæmic women to doubt the causative influences of these conditions as well as that of defective health. This is more certain in single or nulliparous women than in those who have borne children, in whom old laceration of the cervix may be the source of the trouble, or chronic hyperæmia from displacements, the friction of pessaries, sexual excesses, and so forth may be more probably etiological factors. Corporeal endometritis may spread to the cervix from above; while masturbation is not only the cause of physical ill-health, but the means of introducing septic and irritating matter into the vagina, and setting up chronic disease of the cervix. Indeed, anything that affects the general well-being of the patient, such as menstrual chill, bad hygienic surroundings and over-fatigue, may cause or increase cervical catarrh, while the septic and other dangers of childbed are important etiologically, as is everything that can introduce septic matter into the genital tract or lower its normal resistance.

The subject of endocervicitis may complain of local or general symptons, and even when questioned on the former may not have any very pronounced or suggestive symptoms to describe. If she does not suspect the existence of some uterine disease, she is not likely to draw our attention to it. Backache, e.g., is unhappily too common a complaint with women for them to attach any particular significance to it. So with "whites." Present from time to time near the menstrual period, or from some passing congestion of the mucosa, leucorrhœa insensibly persists or increases insidiously till the patient ceases to consider it as abnormal. She may even deny the presence of leucorrhœa, or only recall (as so often happens) after the diagnosis has been made, that she had suffered from a more or less profuse discharge of the nature of gum water, egg albumin, or starch; that it adhered to the linen and stiffened it. But though patients are apt to be indefinite as to the quality and quantity of a leucorrhœa, they generally complain of a weak back, of a feeling of lassitude and ill-health, some pain or dragging in the loins, or a sensation of bearing down in the pelvis that is increased by fatigue or exertion. They have an indifferent appetite, too, and some dyspeptic troubles. If such a patient—especially, though not necessarily, a multipara—consults you, uterine trouble may be suspected and a physical examination is called for.

It will be well to remember first, however, that some of the secondary symptoms may be so severe as to obscure the primary affection, not only for the patient, but for the unwary practitioner. The general ill-health in a neurotic subject may amount to disturbance of an unstable nervous equilibrium so severe as to result in "nervousness," palpitations, neuralgias anywhere and everywhere, flatulence and reflex irritation of the stomach that may be manifested by nausea or even vomiting, while headaches are common. Some women complain a good deal of irritation and tenderness of the nipples, aggravated towards the menstrual period. The patient does not usually suffer from bladder trouble; that is more common in acute conditions, the more so if gonorrhœal in origin. And these symptoms are not those of the disease entirely; they are symptoms of the patient, and vary with the personality, as in all varieties of disease, but especially in uterine disease.

Sterility may be the occasion of your being consulted. A woman has been married some years without becoming pregnant, and examination reveals cervical catarrh as a probable cause. I am disposed to believe that the excessive secretion damages the vitality of the spermatozoa rather than that the mechanical obstruction of the plug of mucus is the reason for sterility in these cases, but there can be no doubt that chronic cervical catarrh is a very common cause of sterility. And what is the nature of this secretion? It is not readily noticed, being scanty in quantity as a rule, but patients describe it as being whitish, like gum water or white of egg, when they observe it at all. It may be left to a physical examination to recognise it, when it will be seen as a plug of opalescent, viscid matter filling the cervical canal. Twist a little cotton wool on a probe or game skewer, and turn it round in the cervix, and you may draw out a string of this mucus to some length without breaking. Cervical discharge is much more easily overlooked than that from the endometrium, and cannot be excluded without a physical examination.

Any pain complained of is usually referred to the loins or the sacral region, but patients not unusually insist rather on secondary pains and neuralgia or backache is only elicited on questioning. Indeed, the constitutional condition is very often most prominent in these cases, and the surgeon must not be led away from the origin of general symptoms. Menstruation is not markedly altered. It may be scanty, but excess in quantity or dysmenorrhœa are more commonly associated with such complications as endometritis. So with dyspareunia, which, unless caused by hyperplasia of the cervix, more generally results from parametritis accompanying the cervical catarrh, and more urgently calling for treatment.

Physical examination discloses characteristic signs. On introducing the finger into the vagina we may note in nulliparæ a small external os, a cervix distended with retained secretion and elliptical in shape, with some tenderness on pressure opposite the internal os. The lips of the os may in multiparæ be found lacerated, a common cause of cervical catarrh; or feel velvety from the presence of catarrhal patches, or rough from retention cysts formed in Nabothian follicles—little nodular projections the size of a split pea—or the cervix may be converted into a cystic mass.

Put the patient in the Sims position and insert his speculum, and these things are seen. If the cervix is lacerated, it will be necessary to seize it with volsellæ or tenacula, and roll outwards its lips, in order to see the extent of the laceration. By rolling them inwards again you will be able to estimate the true size and shape of the os. Its lips may be much enlarged or marked by polypoidal projections and numerous ovula Nabothii. We have seen that the tenacious, stringy secretion may be removed and examined on a dressed probe. In bad cases the lips of the os may be so thickened and distorted that it is difficult to exclude malignancy, at all events in its early stages. But if you are not satisfied as to this on noting that they are not pliable, and do not bleed freely on examination, and that fixation of the uterus is absent, it is a simple matter to swab the cervix and vagina with an antiseptic, and snip a piece off with scissors for microscopical examination. As to the leucorrhœa, you can see that it is uterine in origin, and, except in the worst cases, where it may be yellow or greenish, of the characters described; while if it come from the body of the uterus there are other symptoms, e.g., menorrhagia, a rough and enlarged uterine cavity, that will accompany it. Syphilis is very rare, and would be diagnosed by a specific history and symptoms.

